# The Role of PDGFs and PDGFRs in Colorectal Cancer

**DOI:** 10.1155/2017/4708076

**Published:** 2017-01-10

**Authors:** Roberta M. Manzat Saplacan, Loredana Balacescu, Claudia Gherman, Romeo I. Chira, Anca Craiu, Petru A. Mircea, Cosmin Lisencu, Ovidiu Balacescu

**Affiliations:** ^1^First Medical Clinic, University of Medicine and Pharmacy “Iuliu Hatieganu” Cluj-Napoca, 3-5 Clinicilor Street, 400006 Cluj-Napoca, Romania; ^2^Department of Functional Genomics, Proteomics and Experimental Pathology, The Oncology Institute “Prof. Dr. Ion Chiricuta”, 34-36 Republicii Street, 400015 Cluj-Napoca, Romania; ^3^The Regional Institute of Gastroenterology and Hepatology “Prof. Dr. Octavian Fodor” Cluj-Napoca, 5 Constanta Street, 400158 Cluj-Napoca, Romania; ^4^Department of Surgical and Gynecological Oncology, University of Medicine and Pharmacy “Iuliu Hatieganu”, 34-36 Republicii Street, 400015 Cluj-Napoca, Romania; ^5^Department of Surgery, The Oncology Institute “Prof. Dr. Ion Chiricuta”, 34-36 Republicii Street, 400015 Cluj-Napoca, Romania

## Abstract

*Introduction.* Colorectal cancer (CRC) is an important cause of morbidity and mortality worldwide. Angiogenesis was reported as one important mechanism activated in colorectal carcinogenesis. Tumor microenvironment associated angiogenesis involves a large spectrum of signaling molecules and deciphering their role in colorectal carcinogenesis still represents a major challenge. The aim of our study is to point out the diagnosis and prediction role of PDGF family and their receptors in colorectal carcinogenesis.* Material and Methods.* A systematic search in Medline and PubMed for studies reporting the role of platelet-derived growth factors (PDGFs) and their receptors (PDGFRs) in tumor biology related to CRC was made.* Results.* PDGFs are important growth factors for normal tissue growth and division, with an important role in blood vessel formation. PDGFs/PDGFRs signaling pathway has been demonstrated to be involved in angiogenesis mainly by targeting pericytes and vascular smooth muscle cells. High levels of PDGF-BB were reported in CRC patients compared to those with adenomas, while elevated levels of PDGFR *α*/*β* in the stroma of CRC patients were correlated with invasion and metastasis. Moreover, PDGF-AB and PDGF-C were correlated with early diagnosis, cancer grading, and metastatic disease.* Conclusions.* Both PDGFs and PDGFRs families play an important role in colorectal carcinogenesis and could be considered to be investigated as useful biomarkers both for diagnosis and treatment of CRC.

## 1. Introduction

Colorectal cancer (CRC) is an important cause of morbidity and mortality worldwide, especially due to the deficiency of early detection reliable biomarkers. CRC develops via a complex process from a low-grade dysplasia adenoma to a high-grade dysplasia adenoma and finally to adenocarcinoma. The adenoma-carcinoma transition is recognized as playing a considerable role in colorectal tumorigenesis, and colorectal adenomas are seen as precursor lesions of CRC. Progression through this process is characterized by a complex interaction between environmental carcinogens, genetic mutations, and the host immune system, eventually leading to the uncontrolled growth of modified cells [1].

Nowadays, colonoscopy and fecal occult blood tests (FOBT) are screening methods currently used to diagnose the patients with CRC. However, the invasive character of colonoscopy is an element which further limits its application. Despite the fact that FOBT is a simple, affordable, and noninvasive test, it has a poor sensitivity for the early detection of CRC [2]. Unlike many other tumors, CRC is a preventable and possibly treatable disease if high-grade dysplasia adenomas and early stage tumors are diagnosed and removed. Hence, new biomarkers are needed for diagnosing the precursor lesions and early stages of CRC. Molecular tests are assumed to be better than current screening methods and provide specific details about the tumor progression. Intensive research efforts tracing the identification of noninvasive biomarkers for early diagnosis of CRC in blood and stool are ongoing. Tumor microenvironment (TME) represents a new hallmark of cancer [3] and includes complex cooperation between tumor cells with stroma, immune cells, and endothelial cells. Moreover, the presence of inflammatory cells and inflammatory mediators such as chemokines and cytokines related to TME facilitate tumor progression, including CRC [4]. Sometimes, a single cytokine (e.g., growth factor) can activate signals of complex molecular cascades resulting in tumor progression and development. In line with this view, tumor angiogenesis and vasculature remodeling represent two important mechanisms activated in CRC. Although some of the molecules involved in these mechanisms such as VEGF (the vascular endothelial growth factor), FGF (fibroblast growth factor), and TGF*β* are well characterized, deciphering the role of other molecules such as PDGF (platelet-derived growth factor) is still challenging. Several studies highlighted that PDGFs/PDGFRs are often expressed in diverse tumors and their expression is correlated with tumor growth and spread, therapy resistance, and poor clinical results [5]. Understanding the role of PDGFs/PDGFRs in colorectal carcinogenesis may provide new data for diagnosis and prognosis of CRC and for the discovery of future new therapeutic strategies.

In this review we discuss the role of platelet-derived growth factors (PDGFs) and their receptors (PDGFRs) in tumor biology related to CRC.

## 2. The Role of PDGFs and PDGFRs in Colorectal Carcinogenesis

### 2.1. Tumor Angiogenesis

Angiogenesis is a well-regulated mechanism which in normal conditions is characterized by a proportionate equilibrium between pro- and antiangiogenic factors as well as between multiple signaling pathways [6]. In the case of appearance of malignancy, there is a disruption of this equilibrium between pro- and antiangiogenic factors, known as “angiogenic switch” given by the enhancement of nutrient supply essential for tumor growth [7]. Tumor angiogenesis is an important process involved in the development and spread of CRC [8]. During tumor development, oxygen distribution is scarce, and tumors become progressively influenced by their intrinsic blood supply. Proangiogenic proteins of TME determine the proliferation of endothelial cells and the growth of the tumor vasculature.

Currently, members of the VEGF family and their receptors have been recognized as mediators of angiogenesis. The binding of VEGF to their receptors situated on endothelial cells induces chain reactions that are mostly mediated by MAP kinase and PI3K/Akt/mTOR [9]. During tumor progression, in hypoxic condition, key angiogenic factors such as VEGF, PDGF, FGF, and TGF*β* are under the control of HIF-1 [10, 11]. The importance of VEGF, FGF, and TGF*β* in tumor angiogenesis including in the colon carcinogenesis has been extensively analyzed in the literature [12–14]; therefore, in this review, we will focus on the role of PDGFs/PDGFRs in the CRC pathology. The roles of PDGF signaling in tumor angiogenesis imply pericyte recruitment to vessels, stimulation of proangiogenic factors, endothelial cell proliferation, migration, and promotion of lymphatic angiogenesis and further lymphatic metastasis [15–20].

### 2.2. PDGF Isoforms and Their Receptors

**Figure 1:**
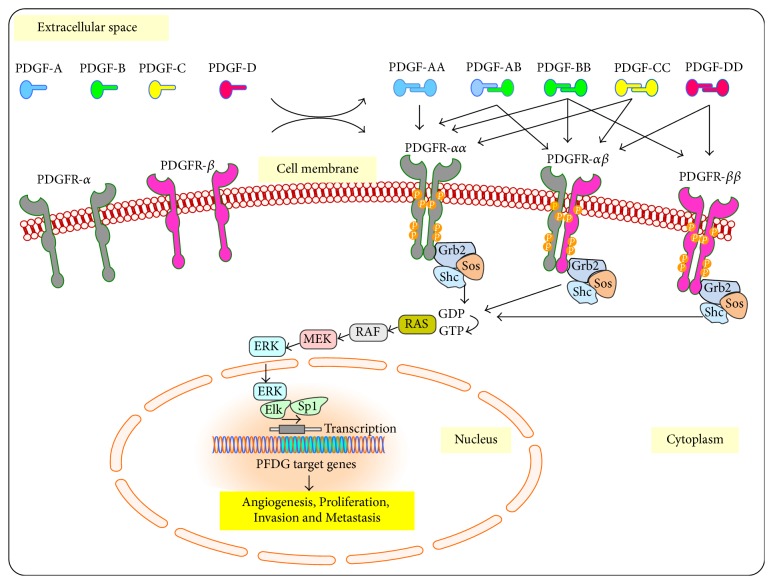
The family of platelet-derived growth factors (PDGFs) and their receptors (PDGFRs) and their functions. Five specific ligands isoforms (PDGF-AA, PDGF-AB, PDGF-BB, PDGF-CC, and PDGF-DD) interact with three of their receptors dimers (PDGFR-*αα*, PDGFR-*αβ*, and PDGFR-*ββ*) and initiate a complex MAP-kinase signaling cascade that activates specific genes involved in angiogenesis, proliferation, invasion, and metastasis.

As mentioned above, the function of VEGF in angiogenesis is accompanied by PDGFs. The PDGFs in their monomeric form are inactive and include four different polypeptide chains (PDGF-A, PDGF-B, PDGF-C, and PDGF-D). PDGFs become active and produce their biological effects through dimerization by binding of monomeric forms by amino acid disulfide bonds (Figure 1). Till now, four homodimers, including PDGF-AA, PDGF-BB, PDGF-CC, and PDGF-DD, and one heterodimer, PDGF-AB, have been described [21, 22]. These PDGF isoforms produce their cellular effects by specific binding to homodimeric and heterodimeric PDGF receptors [23]. Thus, homodimeric PDGFR-*αα* is activated by all PDGF ligands except PDGF-DD while heterodimeric PDGF-*αβ* is activated by all PDGF isoforms except PDGF-AA. Activation of PDGFR-*ββ* occurs only by binding to PDGF-BB and PDGF-DD [5]. It is difficult to separate the signaling pathways and biological functions mediated by PDGFR-*αα* and PDGFR-*ββ* homodimers from that of PDGFR-*αβ* heterodimer. Therefore, the activated receptors are responsible for initiating a complex Ras/MAP-kinase signaling cascade by stimulating downstream effectors such as Grb2/SOS, PI3K/AKT/mTOR, JNK, GAP, and STATs pathways, which are responsible for transcription of PDGF target genes [21, 24].

### 2.3. Normal Functions of the PDGFR/PDGF System

The PDGFR/PDGF system has an important significance in embryogenesis, mainly embryonic growth of blood vessels, and organogenesis; in adults, its physiological role involves tissue repair and wound healing [21]. PDGF isoforms are produced by endothelial or epithelial cells and are implicated in the growth of mesenchymal cell [17]. Signaling through PDGFR-*α* is necessary for the development of the lung, intestinal villus, and facial skeleton, as well as for hair follicle morphogenesis, spermatogenesis oligodendrocytes, and astrocytes [25–28]. On the other hand, signaling through PDGFR-*β* is essential for maturation of blood vessels, white adipocytes, and kidneys [24–27].

### 2.4. Altered Functions of PDGF/PDGFRs Correlated with Cancer

The oncogenic mutations of PDGFRs and overexpression of PDGF/PDGFRs members are implicated in cancers. Alterations of the PDGF/PDGFR genes such as activating mutations [29], translocation and amplification [30, 31], and translocation and deletion [29, 32, 33] can lead to an increased PDGFR signaling. Pathogenic mutations of the PDGFRs appear particularly in the intracellular domain, at kinase and juxtamembrane domain [5, 24, 33, 34], but recently pathogenic extracellular alterations of PDGFR-*α* have been reported as well [35, 36]. All these pathogenic alterations have been notably associated with poor outcomes of cancers, suggesting their role as prognostic biomarkers. PDGFs and their receptors have attracted the interest of researchers given that they are often overexpressed in different tumors including CRC, and their expression is associated with diagnosis [37], tumor growth [38, 39], drug resistance [12], invasion, and poor survival [40, 41].

## 3. The Role of PDGFRs in Colorectal Carcinogenesis

The alteration of signaling by PDGFRs family has an important role in colorectal carcinogenesis. Generally, CRC is associated with overexpression of PDGFRs in tumors and/or tumor-associated stromal cells [5, 42–45]. The overexpression of PDGFRs in CRC is related to angiogenesis, invasion, and metastasis as well as poor survival and target associated therapy (Table 1). In a previous study Wehler et al. [46] reported that the majority of CRC specimens investigated in 99 cohort patients revealed a PDGFR-*α*/*β* expression and this expression was significantly correlated with lymphatic dissemination and metastatic disease. Furthermore, Steller et al. [44] found that high PDGFR-*β* expression in CRC tumors could be associated with the occurrence of metastasis. In this regard, Estevez-Garcia et al. [45] investigated for the first time the incidence of genetic polymorphisms in these receptors and its possible clinical associations with CRC progression. The authors investigated VEGFR2, PDGFR-*α*, and PDGFR-*β* tyrosine-kinase (TK) domain for genetic variants both in CRC cell lines and in CRC samples of 92 patients; four genetic variants were identified and the G-allele genotype of PDGFR-*β* exon 19 SNP (rs246395) was frequently found (58%) in CRC specimens, also being correlated with increased PDGF pathway activation and poor survival. Several studies have investigated the role of PDGFR overexpression in conjunction with CRC treatment response. Erben et al. [47] showed significantly increased PDGFR-*β* mRNA expression in locally advanced rectal cancers compared with the normal tissue. Moreover, Kitadai et al. [40] demonstrated that the tumor-associated microvasculature in human CRC tissues contained multiple pericytes which can preserve endothelial cells from antiangiogenic treatment; therefore, inhibition of PDGFR activation combined with other antiangiogenic composites may produce therapeutic effects.

## 4. The Role of PDGF-AB in Colorectal Carcinogenesis

**Figure 2:**
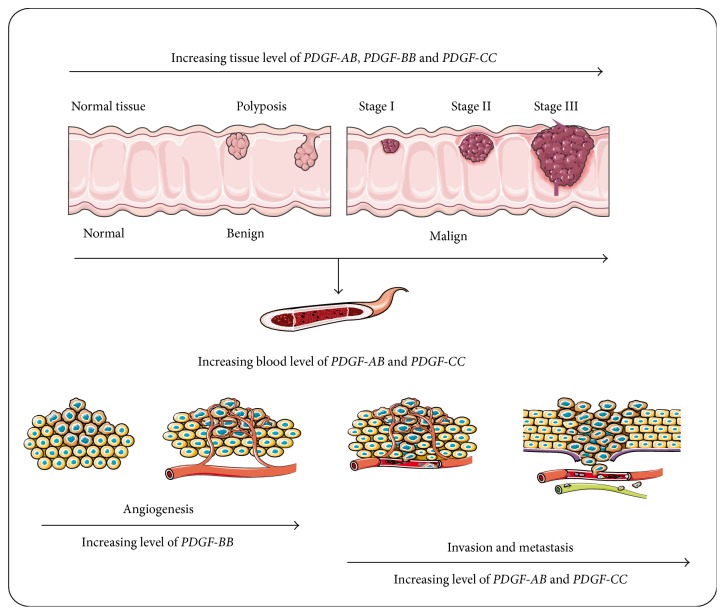
The role of PDGFs ligands (PDGF-AB, PDGF-BB, and PDGF-CC) in colorectal carcinogenesis. Although all three ligands are related to colorectal tissues tumors, only PDGF-AB and PDGF-CC present increased blood levels correlated with tumor stages. An increased level of PDGF-BB is correlated to colorectal tumor angiogenesis, while increasing levels of PDGF-AB and PDGF-CC are closely related to invasion and metastasis.

PDGF-AB is an important molecule that regulates the migration and proliferation of different cells, including CRC cells [48]. Mantur et al. [49] reported that not only the presence of CRC, but also the stage of the disease was correlated with the blood levels of PDGF-AB, which were significantly increased compared to the control group. Similar results were also obtained by Yu et al. [48] who demonstrated that blood levels of PDGF-AB have been correlated with cancer grading, suggesting their role in metastasis formation. In line with this data, and because of association between tissues and blood levels of PDGF-AB, the measurement of PDGF-AB blood expression could be considered for early noninvasive CRC assessment as well as for CRC progression. Moreover, a high level of blood concentration of PDGF-AB might be a significant parameter of the recurrence of CRC (Figure 2). The benefit of tracing the changes in dynamics of PDGF-AB blood levels is the fact that venous blood is a biological material which is easier to obtain than the biopsy of tumor tissues, and its sampling is less invasive.

## 5. The Role of PDGF-BB in Colorectal Carcinogenesis

The discovery that PDGF-B has a homology structure with simian sarcoma virus oncogene (v-sis) and because of its Ras/MAP-kinase signaling that led to transcription of target genes with role in cell survival, proliferation, invasion, and metastasis made PDGF-B to be considered an important oncogene with role in cancer development by PDGF/PDGFR signaling pathways [50].

There is evidence that PDGF-BB modulates angiogenesis in a paracrine manner by inducing blood vessel formation [51] and by stimulating the endothelial cells, by activating other angiogenic factors such as VEGF and FGF and recruiting endothelial precursor cells to angiogenic vessels [5]. One of the first evidence highlighting the role of PDGF-B in colorectal carcinogenesis was mentioned by Ito's et al. [52], who demonstrated the expression of multiloops of growth factors, including PDGF-B, in human CRC cell lines. Since then, other reports have described the role of PDGF-BB in colorectal carcinogenesis. Kitadai et al. [40] revealed that the expression of PDGF-BB proteins was overexpressed at moderate and high level regardless of Duke's stages, concurrent with overexpression of PDGFR-*β* on stromal cells. The association of PDGF-BB with tumor staging was also demonstrated by Ionescu et al. [53] which found a higher level of PDGF-BB in CRC stage Duke B compared with those in stage Duke C and Duke D.

More recently, it was demonstrated that the role of PDGF-BB in CRC is related to increasing pericytes within tumors [54]. Pericytes regulate vascular function, including vessel diameter and vascular permeability, ensure the mechanical support and the stability of the vessel wall, and sustain endothelial cell survival [55]. PDGFR-*β* is the receptor particularly expressed on vascular smooth muscle cells (VSMCs) and pericytes and is the primordial receptor for the PDGF-BB homodimer [56]. Considering their roles in CRC developing, Belizon et al. [57] have evaluated the plasma level of PDGF-BB as a possible noninvasive biomarker. They identified median significantly higher levels of PDGF-BB in patients with CRC tumors compared with those with adenomas, suggesting that PDGF-BB could be evaluated as a biomarker for diagnosis. In conclusion, PDGF-BB is supposed to be implicated in the development of CRC, sustaining angiogenesis by increasing pericytes within tumors.

## 6. The Role of PDGF-CC in Colorectal Carcinogenesis

PDGF-CC is a mitogenic factor, with higher activity than PDGF-AA but comparable with PDGF-AB and PDGF-BB, for cells of mesenchymal origin [58]. Because PDGF-CC has a mitogenic activity comparable to PDGF-AB and PDGF-BB and because of its affinity to both PDGFR-*αα* and PDGFR-*αβ*, it is also considered an important oncogene of PDGFs/PDGFRs signaling pathway. Previous study demonstrated that PDGF-CC promotes angiogenesis, by both PDGFR-*αα* and PDGFR-*αβ* receptors in endothelial cells [59].

There are a few studies that revealed the link between PDGF-CC expression and CRC. Yamauchi et al. [60] showed that the expression of PDGF-C mRNA was significantly higher in tumor tissues than in adjacent noncancerous tissues. Moreover, the authors have revealed that PDGF-CC mRNA was higher in tumor tissues of patients with metastatic disease than those with nonmetastatic disease, suggesting that PDGF-CC could contribute to metastasis in CRC (Figure 2). Furthermore, a significant correlation between PDGF-C protein expression and clinic-pathological features was found, and high levels of PDGF-CC were predictive of recurrence of CRC after curative surgery. PDGF-CC could be a useful biomarker to choose the type of adjuvant chemotherapy in CRC patients after curative surgery; intensive adjuvant chemotherapy could be relevant to CRC patients with high levels of PDGF-CC even if they do not have other common risk factors for recurrence. In addition, a combination of PDGF-C level with TNM classification might ameliorate the prediction of prognosis in CRC patients [60]. Furthermore, it is admitted that PDGF-C acts in a paracrine way to recruit cancer-associated fibroblasts and stimulates angiogenesis and tumor development [61]. Recently, our group [37] had found for the first time a significantly increased expression of PDGF-C in the blood of CRC patients compared to control group. Our findings suggested that the level of PDGF-C peripheral blood might contribute to early diagnosis of CRC, and PDGF-C could be used as a noninvasive biomarker for CRC diagnosis.

## 7. The Role of PDGF-DD in Colorectal Carcinogenesis

PDGF-DD is commonly upregulated in different types of cancers, but its role in CRC was not established yet.

## 8. Antiangiogenic PDGF/PDGFR-Based Therapy

Antiangiogenic therapies that target different signaling pathways at once have been discovered in the hope of improving antitumor efficacy [63]. Angiogenesis development requires close cooperation between several growth factors families and their specific receptors. Understanding the factors/receptors cascade signaling has led to the development of antiangiogenic therapy [64]. The majority of antiangiogenic therapies were developed for VEGFs/VEGFRs families, but due to the need of better control of angiogenesis, other targets including PDGFs/PDGFRs were considered. The knowledge about the implication of PDGFs for proper maturation and function of blood vessels has led to the discovery of various types of antagonists of PDGF signaling. Targeting PDGFRs that primarily act on immature blood vessels has attracted attention as antiangiogenic therapy [62]. Targeting PDGF/PDGFR pathway in malignant diseases that particularly express PDGFRs and PDGFs could improve standard therapies in cancers. Nowadays, there are several studies ongoing to clarify the role for PDGFs/PDGFRs pathways in tumor angiogenesis, in the hope of discovering more potent antiangiogenic therapies that reduce growth, invasion, and metastases of different tumor types.

It seems that the antiangiogenic outcomes of these therapies occur partly via activation of endothelial cell apoptosis, reduced vessel permeability, and decreased blood flow [65–67]. At the present time, multitargeting antiangiogenic agents are in advanced stage of clinical trials which include cediranib, linifanib, dovitinib, lenvatinib, and brivanib agents that inhibit diverse VEGFR, FGFR, and PDGFR family members [63]. Among these antiangiogenic agents, cediranib is the most potent, with a multitude of indications, including CRC, glioblastoma, biliary tract cancer, and ovarian cancer. The antiangiogenic approved agents which inhibit PDGF/PDGFR family are highlighted in Table 2.

## 9. Conclusions

All these observations mentioned above pointed out that PDGFs and PDGFRs are overexpressed in various cancers including CRC. PDGFR-*α* and PDGFR-*β* are involved in tumor angiogenesis and their expression corresponds with tumor growth, invasion, metastasis, and poor survival. The blood level of PDGF-AB and PDGF-CC can be correlated with CRC stages and with early diagnosis and metastatic disease. Further, as an angiogenesis modulator, PDGF-BB level is associated with disease severity. In addition, many reports indicate that PDGFs/PDGFRs have an evident clinical potential in the validation as diagnosis and/or prognosis biomarkers in CRC. This could improve prevention, clinical outcomes, and the prognosis of CRC. Moreover, the use of PDGFs/PDGFRs antagonists in combination with various therapeutic strategies for targeting different molecular drivers seems to be the eventual approach for potent PDGF isoforms and receptors inhibitors in the cancers therapy.

## Figures and Tables

**Table 1:** The role of PDGF/PDGFR families in colorectal carcinogenesis.

PDGF/PDGFR member	Role in CRC	References
PDGF-AB	Correlation with cancer grading	Mantur et al. [49]
	Early diagnosis and cancer grading	Yu et al. [48]

PDGF-BB	Increasing the pericytes within tumors and decreasing the tumor growth	McCarty et al. [54]
	Expression of this protein in CRC patients	Ito et al. [52]
	Correlation with tumor staging	Ionescu et al. [53]
	Biomarker for diagnosis	Belizon et al. [57]

PDGF-C	Metastatic disease	Yamauchi et al. [60]
	Early diagnosis	Manzat-Saplacan et al. [37]

PDGFR-*α*/*β*	Tumor angiogenesis, invasion, metastasis	Song et al. [38]
	Tumor angiogenesis, invasion, metastasis	Kitadai et al. [40]
	Correlation with advanced cancer	Erben et al. [47]
	Targeting PDGFRs as antiangiogenic therapy	Hurwitz et al. [62]
	Lymphatic dissemination/metastatic disease	Wehler et al. [46]
	Metastatic disease	Steller et al. [44]
	Poor survival	Estevez-Garcia et al. [45]

**Table 2:** Agents approved as anti-PDGF/PDGFR.

Drug	Target	Type of cancer
Sorafenib (Nexavar)	VEGFR, *PDGFRs*, FGFR1, KIT, RAF	Metastatic RCC
		Unresectable HCC
Sunitinib (Sutent)	VEGFRs, *PDGFRs*, KIT, FLT-3	Metastatic RCC
		GIST
		Unresectable pancreatic neuroendocrine tumors
Pazopanib (Votrient)	VEGFRs, *PDGFRs*, KIT	Metastatic RCC
		Advanced soft tissue sarcoma
Axitinib (Inlyta)	VEGFRs, *PDGFRs*, KIT	Metastatic RCC
Regorafenib (Stivarga)	VEGFRs, TIE2, *PDGFRs*, RET, KIT, FGFRs	Metastatic CRC
		GIST

RCC, renal cell carcinoma; VEGFR, vascular endothelial growth factor receptor; FGFR, fibroblast growth factor receptor; KIT, v-kit feline sarcoma viral oncogene homolog; RAF, v-raf-1 murine leukemia viral oncogene homolog 1; HCC, hepatocellular carcinoma; FLT-3, fms-related tyrosine kinase 3; GIST, gastrointestinal stromal tumor; RET, ret protooncogene; TIE, tyrosine kinase endothelial.
